# Zinc Ameliorate Oxidative Stress and Hormonal Disturbance Induced by Methomyl, Abamectin, and Their Mixture in Male Rats

**DOI:** 10.3390/toxics5040037

**Published:** 2017-12-03

**Authors:** Sameeh A. Mansour, Mostafa A. Abbassy, Hassan A. Shaldam

**Affiliations:** 1 Environmental Toxicology Research Unit (ETRU), Pesticide Chemistry Department, National Research Centre, Dokki, Giza 12311, Egypt; 2 Department of Pest Control and Environmental Protection, Faculty of Agriculture, Damanhour University, Behira, Egypt; maabbassy@yahoo.com (M.A.A.); Hassan_shaldam@yahoo.com (H.A.S.)

**Keywords:** methomyl, abamectin, methomyl+abamectinmixture, zinc, joint action, amelioration index

## Abstract

Exposure to mixtures of toxicants (e.g., pesticides) is common in real life and a subject of current concern. The present investigation was undertaken to assess some toxicological effects in male rats following exposure to methomyl (MET), abamectin (ABM), and their combination (MET+ABM), and to evaluate the ameliorative effect of zinc co-administration. Three groups of rats were designated for MET, ABM, and the mixture treatments. Three other groups were designated for zinc in conjunction with the pesticides. Additionally, one group received water only (control), and the other represented a positive zinc treatment. The obtained results revealed that MET was acutely more toxic than ABM. The tested pesticides induced significant elevation in lipid peroxidation and catalase levels, while declined the levels of the other tested parameters e.g., Superoxide dismutase (SOD), Glutathione-S-transferase (GST), Glutathione peroxidase (GPx), Glutathione reductase (GR), Cytochrome P_450_ (CYP_450_), testosterone, and thyroxine). Biochemical alterations induced by the mixture were greater than those recorded for each of the individual insecticides. The joint action analysis, based on the obtained biochemical data, revealed the dominance of antagonistic action among MET and ABM. Zinc supplementation achieved noticeable ameliorative effects. It was concluded that zinc may act as a powerful antioxidant, especially in individuals who are occupationally exposed daily to low doses of such pesticides.

## 1. Introduction

Methomyl, *S*-methyl *N*-(methylcarbamoyloxy) thioacetimidate (C_5_H_10_N_2_OS), is an oximecarbamate insecticide produced by DuPont since 1966. It is used widely for controlling insect pests on fruits, vegetables, vines, grains, soybeans, and cotton all over the world [1]. The *N*-methyl carbamate insecticides, such as methomyl, inhibit acetylcholinesterase (ChE) activity, inducing cholinergic overstimulation, and autonomic and neuromuscular dysfunction. At high doses, it causes coma and death [2]. Methomyl is classified as a highly hazardous (class 1B) compound by heWHO [3]. It has been reported that methomyl is capable of inducing oxidative damage and lipid peroxidation in vitro in rat erythrocytes [4].

Abamectin (ABM) is a natural fermentation product produced by a soil actinomycete, *Streptomyces avemitilis* [5]. It is a mixture of avermectin B1a (80%) and avermectin B1b (20%); differing by a single methylene group. Abamectin is being used in several countries for pest control in livestock and in agriculture as a nematicide and insecticide [6]. This product is highly toxic to mammals and has caused the death of 57 calves over four years due to misuse [7]. The avermectinsincrease the membrane conductance to chloride ions and, thus, block electrical activity in nerves and muscles. The target for abamectins involves the g-aminobutyric acid (GABA) receptor in the peripheral nervous system [8].

Exposure to a cocktail of pesticides, including MET and ABM, from consumption of vegetables and fruits containing their residues is possible. Several residue monitoring studies revealed presence of residues of MET and ABM in a variety of field crops. In a large Brazilian monitoring program for pesticide residues, both MET and ABM were reported among other pesticides in samples of fruits, vegetables, and rice [9].

Owing to the frequent application of MET and ABM with other pesticides (e.g., diniconazole and phenthoate) under greenhouse conditions in Egypt, the pre-harvest intervals (PHI) were determined to be 21 days for MET and phenthoate, 10 days for diniconazole, and three days for ABM in pepper fruits sprayed with the above-mentioned pesticides [10]. Out of 215 pesticides analyzed in 177 samples of fruits collected from different local markets in Egypt, residues of MET were found 0.01, 0.03, 0.09, and 0.01 mg/kg, respectively, in apple, apricot, grape, and strawberry. It seemed that residues of ABM were either undetected or not included in the analytical monitoring program [11]. Recently, Radwan et al. [12] detected residues of 71 pesticides on green beans, and ABM and MET were found at mean concentrations of 0.016 and 46.47 mg/kg, respectively. Such reports support the possibility of food contamination by both MET and ABM and, thus, exposure to their combination in the diet.

It has long been recognized that induction of oxidative stress by the formation of reactive oxygen species (ROS) is one of the main mechanisms of different xenobiotics (e.g., pesticides, heavy metals, and chemotherapeutic agents) that may generate ROS [13]. Several substances, including essential mineral elements, such as Zn and Se, acted as antioxidants against chlorpyrifos [14,15] and methomyl [16]. Hence, they were used to alleviate toxic hazards of pesticide-induced oxidative stress in experimental animals. In a parallel investigation, we studied the protective effect of zinc, especially at high lethal doses of MET [17]. To the best of our knowledge, the literature offers a single study dealing with the ameliorative effect of vitamins C and E against abamectin toxicity in the liver, kidney, and testes of male albino rats [18].

On the other hand, studies on ABM toxicity in rats is relatively rare and mainly directed to assess its hepato-renal toxicity [18,19,20], with a noticeable lack of the effects on antioxidant status of experimental animals. Additionally, studies on ABM in combination with other pesticides are very limited [21,22].

The present study was conducted to test the oxidative stress induced by ABM, MET, and their mixture, and to assess the ameliorative effect of zinc supplementation in conjunction with each of the individual pesticides and their mixture, using male rats as a test model organism. Based on the estimated biochemical parameters, the joint action will be analyzed. Studies on hepato-renal enzymes will be published elsewhere.

## 2. Materials and Methods

### 2.1. Chemicals

Methomyl, an oximecarbamate insecticide (methyl *N*-[[(methylamino) carbonyl] oxy] ethanimidothioate; IUPAC), was obtained from BASIF Limited Co., Cairo, Egypt, as Nudrin^®^ (90% SP). Abamectin, a biopesticide (molecular weight: 873.1 (avermectin B1a); 860.1 (avermectin B1b); empirical formula: C_48_H_72_O_14_ (80% avermectin B1a); C_47_H_70_O_14_ (20% avermectin B1b), was obtained from Al-Mustafa Triad Co., Cairo, Egypt, asVabcomic^®^ (1.8% EC). Zinc chloride (ZnCl_2_) powder (molecular weight: 136.29), a product of Oxford Laboratory, was purchased from Neminath Industrial Estate No. 6 Navghar, Vasai East, Thane.

### 2.2. Reagents (Diagnostic Kits)

Diagnostic kits used in the present study were obtained from Biodiagnostic Co., Giza, Egypt. These were oxidative/anti-oxidative biomarkers: lipid peroxidation (LPO), superoxide dismutase (SOD), catalase (CAT), glutathione peroxidase (GPx), glutathione reductase (GR), glutatione-S-transferase (GST), cytochrome P_450_ (CYP_450_), and hormonal biomarkers: testosterone (T) and tetraiodothyronine-thyroxine (T_4_).

### 2.3. Animals

Healthy male albino rats of the Wister strain (*Rattusnorvegicus)*, 60 days of age and with average weights of 110 ± 20 g, were obtained from the Animal Breeding House of the National Research Centre (NRC), Dokki, Cairo, Egypt, and maintained in clean plastic cages in the laboratory animal room (23 ± 2 °C) on a standard pellet diet and had free access to water in daily dark/light cycle of 12/12 h. Rats were acclimatized for one week prior to experimentation. The experimental work on rats was performed with the approval of the Animal Care and Experimental Committee, College of Agriculture in Damanhur, Egypt, and according to the guidance for care and use of laboratory animals [23].

### 2.4. Determination of Oral LD_50_ for the Tested Insecticides

Preliminary tests were carried out to determine the median lethal dose (LD_50_) for commercial formulations of methomyl (MET) and abamectin (ABM) on male rats. For each insecticide, three doses were prepared in water based on active ingredient (a.i.) contents (e.g., 10, 20, and 30 mg/kg b.w. for methomyl; and 10, 20, and 30 mg/kg b.w. for abamectin). Four rats were used for each tested dose, in addition to four rats given water only, serving as a control group. Dosing was performed by gavages with 0.5 mL solutions. The 24h-LD_50_ values were estimated according to Finney [24]. Based on the obtained LD_50_ values, the equivalent to the 1/10 was used in the present study (e.g., 2.03 and 1.7 mg/kg b.w., respectively, from MET and ABM).

### 2.5. Dosing and Treatments

A total of 64 male rats were divided into eight groups (Gs), each contained eight animals. G1 (Cont) received water free of any pesticide and served as negative control. G2 (Zn; positive control) administered ZnCl_2_ in drinking water at a concentration of 227 mg/L (as Zn) according to Goel et al. [25]. G3 (MET) and G4 (ABM) were orally administered 2.03 and 1.7 mg/kg b.w. of MET and ABM, respectively. G5 (MET+ABM) was given both insecticides at their respective doses singly. Groups 6, 7, and 8 were respectively, administered MET, ABM, and MET+ABM by oral gavages in addition to Zn in drinking water. The insecticides were given in 48 h intervals; zinc solution was introduced in sufficient amounts one day after another. Water was permitted *ad libitum* for all groups. The experimental duration was extended up to 42 days, and the doses of insecticides were adjusted weekly according to changes in body weights of the tested animals. Observations on animals were performed one day after another to record any treatment-related clinical signs of toxicity.

### 2.6. Blood and Organs Collection

At the end of the experimental duration, blood samples were withdrawn from the animals under ether anesthesia by puncturing the retro orbital venous plexus with a fine sterilized glass capillary. Blood was collected into heparinized glass tubes to separate plasma and left for 20 min at room temperature, then centrifuged at 3000 rpm (600× *g*) for 10 min using a BOECO centrifuge model C-28, Germany, to separate the plasma. The plasma samples were kept in a deep freezer (−20 °C) until analysis within one week. Finally, the rats were sacrificed by decapitation. Liver, kidneys, and testes were quickly removed, weighed individually, and preserved in 10% formalin saline for further histological studies in another work.

### 2.7. Biochemical Measurements

Enzymatic analyses were measured on Jenway 6305 UV/VIS Spectrophotometer at the specified wavelengths. Hormonal determinations were carried out by using enzyme linked immuno sorbent assay (ELISA; GmbH model Jupiter). The analyses were carried out in accordance to the pamphlet instructions given by the manufacturers, and in light of the published methods.

### 2.8. Statistics

All the obtained data were statistically analyzed using Statistical Analysis (SAS) Software Program (V-2000, SAS Institute Inc., Cary, NC, USA, 2016). Data were analyzed as factorial arrangements of the kind of emulsifying and storage period in a complete randomized design with three replicates. Comparisons among the means of different treatments were achieved by using the least significant difference procedure (LSD) at *p* ≤ 0.05 and *p* ≤ 0.01 levels.

### 2.9. Joint Action Analysis

According to Mansour and Refaie [21], the type of interaction between pairs of toxicants in terms of Interaction Index (I.I.) was estimated using the following formula:

(1) $$I.I.=\frac{M\;\mathrm{value}+C\;\mathrm{value}}{A_{1}\;\mathrm{value}+A_{2}\;\mathrm{value}}$$

where M, C, A_1_, and A_2_ represent the mean values obtained from the biochemical estimation of a studied parameter: M for the mixture value; A_1_ and A_2_ for the values of the individual compounds in that mixture; and C for the control value.

The rating of the interaction indices is subject to one of three cases as follows:

(i) Case of positive effect (i.e., a significant increase of the concerned biochemical parameters above the control values due to the effect of the individual compounds); where I.I. > 1 means potentiation; I.I. = 1 means additive; I.I. < 1 means antagonism.
(ii) Case of negative effect (i.e., a significant decrease of the concerned biochemical parameters below the control values due to the effect of the individual compounds); where I.I. > 1 means antagonism; I.I. = 1 means additive; I.I. < 1 means potentiation.
(iii) Case of no observed effect: In such a case, it is supposed that treatment with the mixture and each of its individual compounds do not induce statistically significant differences between the values of the measured parameters; and thus control and treatments have nearly the same values. Here, the interaction index (I.I.), if determined, will equal 1 (i.e., a result similar to that of an additive effect). The prior statistical examination for the dataset of a given biochemical measurement would assist the differentiation between anadditive case and that of no effect.

For accuracy, a “safety factor” of ±0.05 is added to the indices values when ranking the joint action. This means that additive effect will be considered as I.I. = 1 ± 0.05 (i.e., 0.95–1.05), and the other categories have to be ranked accordingly [21].

### 2.10. Oxidative Stress and Amelioration Analysis

According to Mansour and Gamet-Payrastre [26], alteration in the levels of biochemical parameters due to pesticide treatments could be determined by calculating the percentage of change in pesticide-treated groups relative to untreated control groups. To estimate how much deviation than normal values due to pesticide treatments:

(2) $$\%\;\mathrm{of}\;\mathrm{change}=\frac{\mathrm{Treatment}\;\mathrm{value}\;-\;\mathrm{Control}\;\mathrm{value}}{\mathrm{Control}\;\mathrm{value}}\times 100$$

On the other hand, the “Amelioration Index” (AI) could be estimated by comparing the results of a given biochemical parameter in the groups of pesticides + antioxidant agent (e.g., Zn, here) with the results of the control groups to assess the ameliorative effect of Zn. As AI approaches 1, the amelioration reaches a high degree of normalization to the control value [26]:

(3) $$\mathrm{Amelioration}\;\mathrm{index}\;\left(\mathrm{AI}\right)=\frac{\mathrm{Treatment}\;\mathrm{value}\;\left(\mathrm{Pesticide}+\mathrm{Zn}\right)}{\mathrm{Control}\;\mathrm{value}}$$

## 3. Results

### 3.1. Acute Oral Toxicity of the Tested Insecticides

The estimated oral LD_50_ for methomyl (MET) and abamectin (ABM) against the used male rats indicated that MET was more toxic (17.0 mg/kg b.w.) than ABM (20.3 mg/kg b.w.). These values were used to calculate the doses used in the present study (i.e., 1/10 LD_50_).

### 3.2. Observations on Signs of Toxicity

Afterten days of insecticidal treatments, signs of tremors, restless, excitation, dizziness and weakness were observed in MET, ABM and the mixture (MET+ABM)-treated groups. These signs were more pronounced in MET and ABMrat groups. There were no treatment-related clinical signs of toxicity noted inpesticide + Zn treatments. During the last week of experimental duration, most of the signs of toxicity had disappeared.

### 3.3. Effect on Antioxidant Enzymes and Lipid Peroxidation

**Figure 1:**
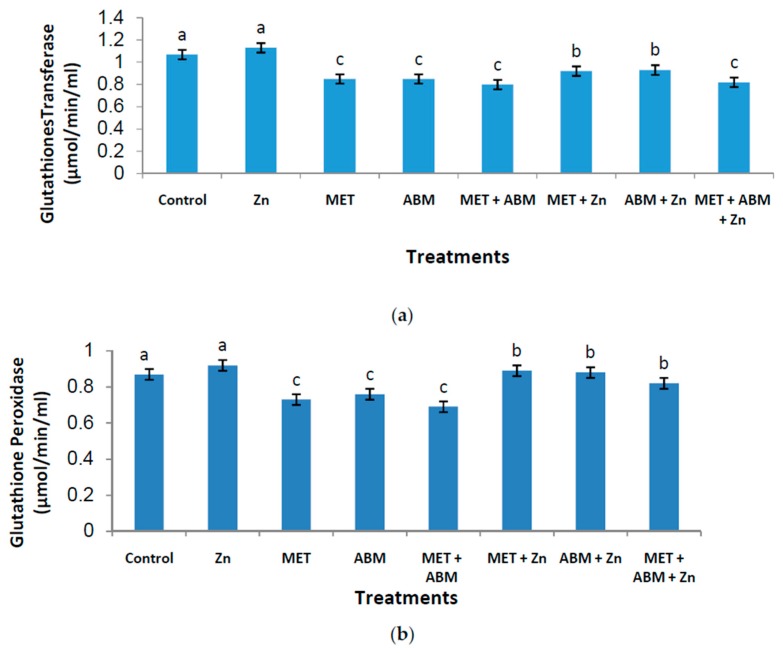

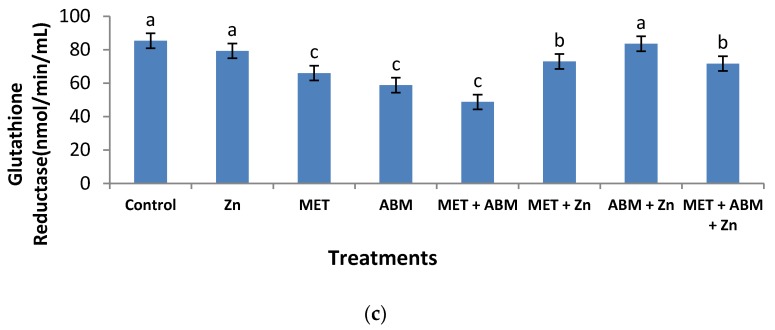
Effect of methomyl (MET), abamectin (ABM), and their mixture (MET+ABM), with and without zinc (Zn), on levels of glutathione-S-transferase(GST), glutathione peroxidase (GPx), and glutathione reductase (GR)in the plasma of male rats. Statistics: bars represent the group means ± SD; *n* = 8. Values of similar superscript letters are not statistically different. Values of superscript “b”are significantly different than those of superscript “a” at *p* ≤ 0.05; Values of superscript “c” are high significantly different than those of superscript “a” at *p* ≤ 0.01. (**a**) = glutathione-S-transferase (GST); (**b**) = glutathione peroxidase (GPx); (**c**) = glutathione reductase (GR).

Six antioxidant enzymes and lipid peroxidations were determined in the plasma of rats treated with MET, ABM, and their mixture (MET+ABM), either with or without zinc administration. Generally, results of control and zinc treatments were of no significant differences. Compared with control result (1.07 µmol/min/mL), the activity of glutathione-S-transferase (GST) recorded 0.85, 0.85, 0.80 and 0.82 µmol/min/mL, respectively for MET, ABM, MET+ABM, and MET+ABM+Zn; indicating highly significant decreases (*p* ≤ 0.01) in the activity of this enzyme. Co-administration of Zn with MET or ABM limited the enzyme decline to some extent (*p* ≤ 0.05), where their estimated values equaled 0.92 and 0.93 µmol/min/mL, respectively (Figure 1a).

The activity of glutathione peroxidase (GPx) (Figure 1b) in the control treatment (0.87 µmol/min/mL) was significantly (*p* ≤ 0.01) higher than the values recorded for MET, ABM, and MET+ABM (0.73, 0.76, and 0.69 µmol/min/mL, respectively). Co-administration of Zn with the individual pesticides or their mixture limited decline of GPx activity to some extent (*p* ≤ 0.05).

Figure 1c illustrates effect of the tested insecticides on the activity of glutathione reductase (GR). The control group recorded 85.37 nmol/min/mL, a value which was significantly higher (*p* ≤ 0.01) than the values recorded for MET, ABM, and MET+ABM (66.01, 58.80, and 48.73 nmol/min/mL, respectively). Co-administration of Zn with ABM normalized the activity of GR (e.g., 83.55 nmol/min/mL).

**Figure 2:**
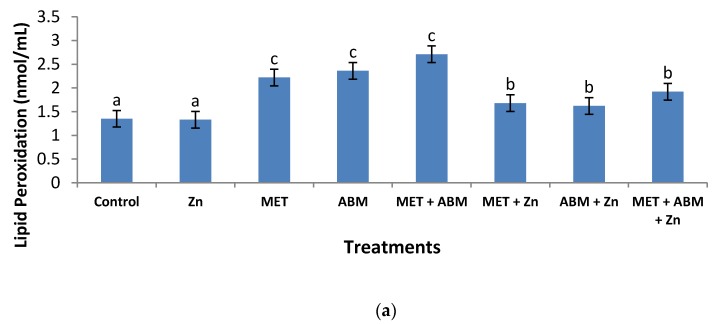

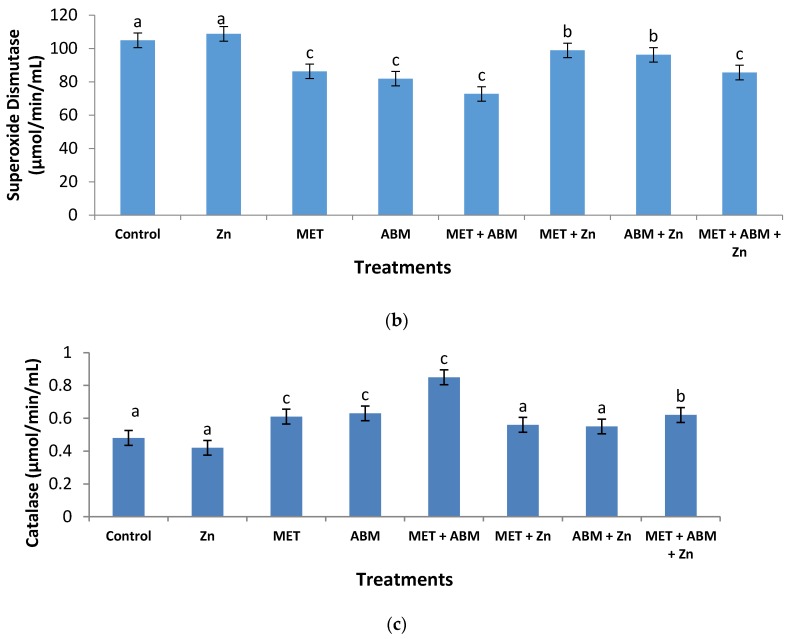
Effect of methomyl (MET), abamectin (ABM), and their mixture (MET+ABM), with and without zinc (Zn), on levels of malondialdehyde (MDA), superoxide dismutase (SOD), and catalase (CAT) in the plasma of male rats. Statistics: narsrepresent the group means ± SD; *n* = 8. Values of similar superscript letters are not statistically different. Values of superscript “b” are significantly different than those of superscript “a” at *p* ≤ 0.05; Values of superscript “c” are high significantly different than those of superscript “a” at *p* ≤ 0.01. (**a**) = malondialdehyde (MDA); (**b**) = superoxide dismutase (SOD); (**c**) = catalase (CAT).

The activity of lipid peroxidation (LPO), in terms of malondialdehyde (MDA), in plasma of control rats was found 1.35 nmol/mL (Figure 2a). MDA recorded high elevation (*p* ≤ 0.01), accounting to 2.22, 2.36, and 2.71 nmol/mL in the rats treated with MET, ABM, and their mixture, respectively. Such high elevation was limited to some extent (*p* ≤ 0.05) by co-administration of Zn.

Contrary to MDA, the activity of superoxide dismutase (SOD) (Figure 2b) showed a highly significant (*p* ≤ 0.01) decline in rats treated with MET, ABM, MET+ABM, and MET+ABM+Zn compared with that recorded for the control (104.87 µmol/min/mL). The greatest decline was attributed to MET treatment (88.23 µmol/min/mL). Administration of Zn with MET or ABM limited such a high decline to some extent (*p* ≤ 0.05).

Catalase activity in the control group recorded 0.48 µmol/min/mL (Figure 2c). In comparison, highly significant elevations (*p* ≤ 0.01) were recorded for MET, ABM, and MET+ABM (e.g., 0.61, 0.63, and 0.85 µmol/min/mL, respectively). Administration of Zn with MET or ABM normalized catalase activity, but failed to achieve a similar result with the mixture (0.85 µmol/min/mL).

**Figure 3:**
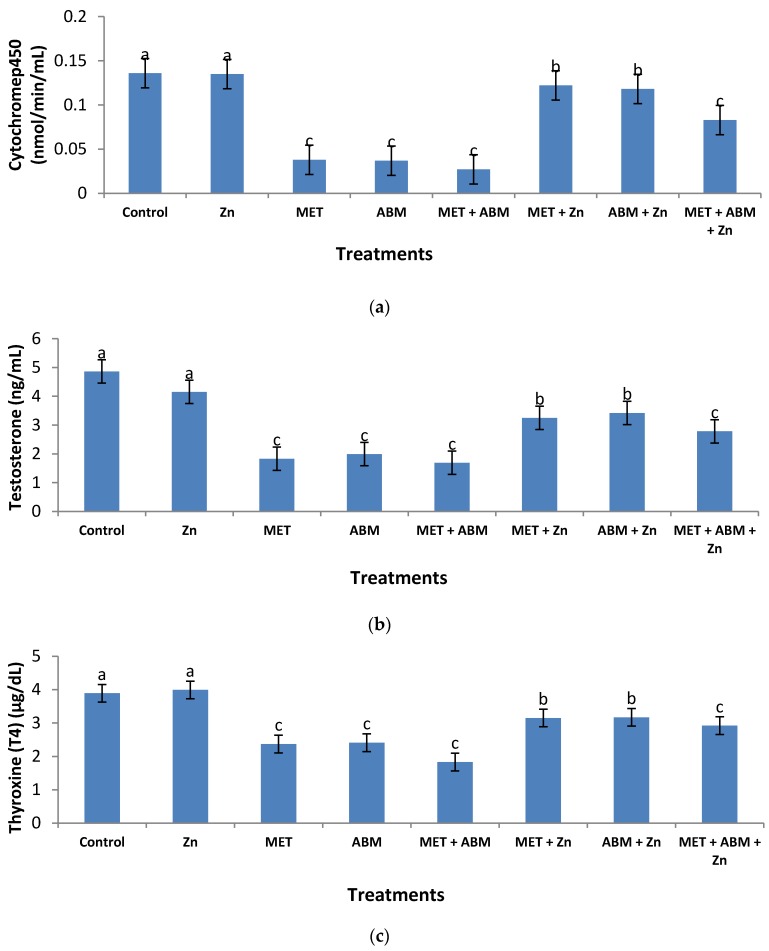
Effect of methomyl (MET), abamectin (ABM), and their mixture (MET+ABM), with and without zinc (Zn), on levels of cytochrome P_450_; testosterone (T), and thyroxine (T_4_) in the plasma of male rats. Statistics: bars represent the group means ± SD; *n* = 8. Values of similar superscript letters are not statistically different. Values of superscript “b” are significantly different than those of superscript “a” at *p* ≤ 0.05; Values of superscript “c” are high significantly different than those of superscript “a” at *p* ≤ 0.01. (**a**) = cytochrome P_450_; (**b**) testosterone (T); (**c**) thyroxine (T_4_).

Figure 3a illustrates cytochrome P_450_ activity in the plasma of male rats treated with the tested pesticides. The control treatment recorded 0.136 nmol/min/mL, a value which was significantly higher (*p* ≤ 0.01) than those recorded for MET (0.038 nmol/min/mL), ABM (0.037 nmol/min/mL), MET+ABM (0.027 nmol/min/mL), and MET+ABM +Zn (0.083 nmol/min/mL). Supplementation of Zn in conjunction with MET or ABM limited the enzyme decline to some extent (*p* ≤ 0.05).

### 3.4. Effect on Testosterone and Thyroxine Hormones

Two hormones, testosterone (T) and thyroxine (T_4_), were determined in the plasma of male rats treated with MET, ABM, and their mixture (MET+ABM), either with or without zinc administration. Testosterone hormone (T) measured in plasma of control rats was found to be 4.86 ng/mL (Figure 3b). Its level was severely reduced (*p* ≤ 0.01) in MET, ABM, MET+ABM, and MET+ABM+Zn treatments, where the estimated values equaled 1.83, 1.99, 1.69, and 2.78 ng/mL, respectively. Co-administration of Zn with MET or ABM limited such decrease (*p* ≤ 0.05) to a noticeable extent.

The hormone, thyroxine (T_4_) showed a similar pattern to that of testosterone where MET, ABM, MET+ABM, and MET+ABM+Zn treatments recorded a severe decline in the level of T_4_ compared with control (3.89 µg/dL). Co-administration of Zn with MET or ABM limited such a decline to a noticeable extent (Figure 3c).

### 3.5. Estimation of Joint Action

Table 1 presents the results of joint action between MET and ABM based on the data of the measured biochemical parameters following treatment of male rats to each of MET and ABM, as well as their binary mixture. Based on the estimated interaction index (I.I.), the mixture interacted potentially against catalase (CAT), and additively against glutathione peroxidase (GPx). The joint action was accounted as antagonistic towards the rest of the tested parameters: malondialdehyde (MDA), superoxide dismutase (SOD), glutathione reductase (GR), glutathion-S-transferase (GST), cytochrome oxidase (CYP_450_), testosterone (T), and thyroxine (T_4_).

### 3.6. Evaluation of Oxidative Stress and Amelioration Effects

The percent of changes in some biochemical parameters in male rats treated with MET, ABM, and the mixture (MET+ABM), and the ameliorative effect of zinc supplementation, are presented in Table 2. In all cases, changes caused by the mixture treatment was higher than those estimated for MET or ABM individually. For example, changes in MDA activity in the mixture treatment was found to be 100.7%, compared with 74.8% and 64.4%, respectively, for ABM and MET treatments. The percent of changes in SOD, CAT, and GR in ABM treatments were higher than those in MET treatments, while the opposite was obtained for GPx, T, and T_4_ in MET treatments. The percent of changes in GST and CYP_450_ in MET and ABM treatments were nearly equal.

The effect of zinc supplementation on minimizing the differences between values of the measured biochemical parameters and their corresponding control values was expressed in terms of the “Amelioration Index” (AI). As shown in Table 2, the AI for GPx equaled 1.02, 1.0, and 0.94, respectively, in the treatments of Zn with MET, ABM, and MET+ABM. The AI value recorded greater than 1.0 for some biochemical parameters and less than 1.0 for other biochemical parameters. The highest AI value (1.42) was entitled to MDA for the mixture treatment+zinc, while the lowest AI value (0.59) was recorded for testosterone in the mixture treatment+zinc.

## 4. Discussion

The estimated acute oral LD_50_ for the used commercial products of MET and ABM to male rats differed slightly and revealed that MET was more toxic than ABM. Both compounds were tested at an equixotic dose (e.g., 1/10 LD_50_), however, they induced varying degrees of alterations in the tested biochemical parameters, as presented above.

In toxicity studies, a variety of specific biochemical parameters are measured to evaluate physiological and metabolic functions which affect target organs and tissue injury [27]. The most widely measured are AST, ALT, ALP for hepatotoxicity, and urea and creatinine for glomerular function [28]. Additionally, cholinesterase (ChE) assay, which is a liver function, is used to assess the cholinergic effects of organophosphorous (OP) and carbamate (CM) pesticides, as well as cholinesterase inhibitors [29]. The literature offers several studies on hepato-renal toxicities of ABM [19,20,30], and MET in rats [14,15,17,19]. However, studies on oxidative stress and antioxidant systems are very limited, especially on ABM, which was reported to induce liver tissue damage in rats that were protected by vitamins C and E [18]. On the other hand, the literature offers no data on the protective effect of zinc against ABM or its combination with MET.

Antioxidant defense plays an important role in the response of organisms to environmental pollutants. Several processes enhance the production of reactive oxygen species (ROS) or deplete the antioxidant defense. Such oxidative stress, if not regulated properly, may lead to damage in DNA, proteins, or lipids. On the other hand, ROS are also beneficial as they play an important role in defense against infectious agents. Hence, a delicate balance between antioxidants and free radical formation is required [31]. Studies have identified ROS as a cause of toxic effects exerted by pesticides [32]. Several substances, including naturally occurring plant oils, vitamins, and essential mineral elements, were used to alleviate toxic hazards of pesticides-induced oxidative stress in experimental animals. In this respect, studies have shown that zinc can protect against oxidative damage caused by certain xenobiotics and, thus, may have antioxidant properties [33]. Our previous studies showed the protective effects of zinc against oxidative stress induced by chlorpyrifos [4] and methomyl [17].

In this respect, it may be convenient to demonstrate the role of the antioxidants of relevance to the present study. Lipid peroxidation (LPO) is a chain reaction between polyunsaturated fatty acids and ROS, yielding lipid peroxides and hydrocarbon polymers. These are extremely toxic to the cells. Peroxidation of polyunsaturated fatty acids and related esters produces malondialdehyde (MDA) as an end product. Therefore, MDA serves as a biomarker of LPO [34]. Catalase is one of the cellular defense mechanisms against cytotoxic oxygen species (H_2_O_2_). However, endogenous (H_2_O_2_) may be reduced to H_2_O either by catalase or glutathione peroxidase, or it may generate the highly-reactive free hydroxyl radical (OH^•^) by the Fenton reaction, which is believed to be mainly responsible for oxidative damage [35]. Glutathione reductase (GR) or reduced glutathione (GSH), is the natural antioxidant of the cell. It has a vital role in the detoxification process by destroying the formed free radicals in the cells. Therefore, deficiency of GR causes greater lipid peroxidation leading to cell damage [36]. Glutathione peroxidase (GPx) is a major defense system against oxidative damage of essential intracellular compounds (e.g., proteins and poly-unsaturated fatty acids); particularly by reducing hydroperoxides to water. Additionally, glutathione-S-transferase (GST) is involved in the detoxification process due to its ability to conjugate GSH with lipid peroxidation products [37]. It is well documented that the antioxidant enzymes, such as SOD, GST, and CAT, act as free radical scavengers by limiting the effects of ROS on the tissues and, thus, they protect the cell from injury [38]. These enzymes work together in order to eliminate ROS and any deviation in the physiological concentrations. The conversion of superoxide radical to H_2_O_2_ is catalyzed by SOD, while CAT converts H_2_O_2_ to water. Therefore, these enzymes have the capability to alleviate the hazards of ROS [35].

In agreement with the results of the present investigation, many studies have reported elevation of lipid peroxidation (LPO) and CAT and a decline of SOD, as well as the group of glutathioneenzymes, following exposure to organophosphorus (OP) pesticides, such as chlorpyrifos [14,15,26]. This was evidenced by the high elevation of thiobarbituric acid reactive substances (TBARS), accompanied with a decrease in the levels of antioxidative stress enzymes (e.g., SOD, CAT and GPx) in the liver, kidney, and spleen [39]. The elevated activity of catalase in insecticide-treated rats in this study may be due to the adaptive response to the generated free radicals indicating the failure of the total antioxidant defense mechanism to protect the tissues from damage caused by free radicals [35].

The cytochrome P_450_ (CYP_450_) enzyme system plays an important role in the bio-activation of cholinesterase inhibitors, such as organophosphorus (OP) pesticides, through catalyzing oxidation of one-atom molecular oxygen into a substrate (e.g., organophosphate) by an electron transport pathway [40]. In this reaction ROS are generated. In the current study, results revealed that the tested pesticides, MET, ABT, and their combination induced significant decreases in the level of CYP_450_. These results are in agreement with those reported by Yamano and Morita [41] and may refer to the inhibition of heme synthesis [42]. Therefore, it has been suggested that the inhibition of cytochrome activity by some pesticides (e.g., OP compounds) may contribute to the development of Parkinson’s disease due to rendering the neurons more sensitive to toxic metabolites of neurotransmitters [43].

Testosterone (T) is the main steroid sex-hormone in male rats. It is secreted by Leydigcells of the testes under the control of complex neuroendocrine interactions [44]. The present findings revealed a pronounced decrease of testosterone levels following exposure of male rats to the tested pesticides. Previous studies reported the decline of this hormone in rats treated with organochlorine (OC) [44] and OP [45] pesticides, as well as ABM [46]. The decrease of the free testosterone level may be a result of direct damage of ABM on Leydig cells in the interstitial tissues, which are the main sites of testicular androgen biosynthesis [44].

Thyroid hormones (e.g., T_4_) might be able to regulate the activities of SOD, CAT, and GPx enzymes in lymphoid organs and skeletal muscles [47]. The role of thyroid hormones in metabolic pathways and antioxidant enzyme activities are documented in many species, such as rats [48] and camels [49]. The results of the present study indicated that the tested insecticides caused decline of thyroxine (T_4_) level in the treated rats. These findings are supported by the results of several investigators who addressed the thyroid inhibitory nature of OP insecticides [50], and a CPF+Pd mixture administered to Wistar rats [51]. The observed decrease in the level of T_4_ in the insecticide-treated groups of rats may refer to some damage in the thyroid gland due to oxidative stress induction and functional impairment of the pituitary-thyroid axis [52].

According to Hassan and Meligi [46], the levels of testosterone and thyroxine hormones were decreased in abamectin-treated groups compared with the control group, and administration of *Eruca sativa* extract showed promising effects against abamectin toxicity-induced disorders of thyroid hormones and impaired testicular functions. On the other hand, a significant decrease in the level of testosterone was observed in the methomyl-intoxicated male rats [53]. Such results support our findings regarding to decline of the levels of the tested hormones.

The results of the present investigation reveal the protective effect of Zn against oxidative stress induced by the tested pesticides. Apart from its direct antioxidant effect by occupying iron and copper binding sites on lipids, proteins, and DNA, zinc also plays a structural role in maintaining the integrity of Cu-Zn-SOD as a cofactor, and in glutathione regulation, which is vital to cellular antioxidant defense [33,54]. To the best of our knowledge, there are no similar studies on MET and ABM mixtures with respect to co-administration with zinc.

Little is known about the impacts of low doses of pesticide mixtures on human health. Major difficulties in this respect are attributed to differences in the levels and exposure periods and frequency of their occurrence, as well as the diversity of active substances and adjuvants used in the formulations of these compounds [55]. Certain pesticides in a mixture may interact chemically, mainly because the metabolism of one chemical can affect the metabolism of the other. Subsequently, mixtures of pesticides can interact additively, synergistically, or antagonistically [56]. Potentiation was reported for the mixture of atrazine+chlorpyrifos+chlorothalonil [57], as well as the mixture of cypermethrin+quinolphos+linuron [58].

The literature offers many publications on chemical mixture research based on biochemical mechanistic studies [59,60,61]. Using biochemical data, Mansour and Refaie [21] analyzed the joint action of six binary mixtures resulting from combination between avermectin, buprofezin, chlorpyrifos, and deltametrhin. Calculations were based on the results of some biochemical parameters (e.g., AST, ALT, ALP, cholesterol, creatinine, urea, and cholinesterase). Out of the 42 studied cases, antagonism was the dominant joint action and represented 66.7% of cases whatever the biochemical criteria used in the assessment process. Additive and potentiation effects represented 26.2% and 7.1% of cases, respectively. Such results parallelthe findings of the present study, which reveal the dominance of antagonistic action, and are supported by Krishnan et al. [59] who concluded that inhibitory, rather than potentiation, was more likely to be observed among interaction of simple mixtures.

In the current investigation, the percent of change in the biochemical parameters following exposure to the tested pesticides indicated how much deviation from normal values occurred. In the majority, changes due to the mixture were higher than changes caused by each of the individual pesticides. On the other hand, the efficiency of zinc to alleviate the oxidative stress exerted by exposure to the tested pesticides resulted in amelioration indices (AI) around 1. Values of AI exceeding 1.0 may refer to either better improvement or negligible experimental errors. The obtained results are supported by our previously published investigations on different pesticides [26,62,63].

## 5. Conclusions

The results of the present study revealed that MET was acutely more toxic than ABM to male rats. The percent of changes in the tested biochemical parameters indicated how much deviation from normal values occurred due to exposure to the pesticides. Treatments with MET and ABM, either individually or in combination, induced significant elevations in lipid peroxidation (in terms of MDA) and catalase levels, while it declined the levels of the other tested parameters (e.g., SOD, GST, GPx, GR, CYP_450_, testosterone, and thyroxine). However, alterations induced by the mixture were greater than those recorded for each of the individual insecticides, and ABM seemed to be more effective than MET. The joint action analysis, based on the obtained biochemical data, revealed the dominance of antagonistic action among MET and ABM. Zinc supplementation in conjunction with the tested pesticides achieved considerable ameliorative effect expressed in terms of the amelioration index (AI), which was close to 1.0, indicating a maximum amelioration effect of zinc in favor of the most-assessed parameters. In conclusion, the study has focused on the joint analysis of the tested mixture and the role of zinc to alleviate oxidative stress exerted by low doses of individual pesticides and their combination, providing novel data of special concern to individuals who are occupationally exposed daily to low doses of such toxicants.

## 6. Recommendations

Since this study was carried out on rats, more implementation regarding human health, pesticide management, agriculture, industry, and regulatory agencies may take into consideration the following proposals:

➢ The use of pesticide mixtures of “antagonistic action” should be encouraged to protect public health.
➢ Most current global environmental agencies derive and regulate their pesticide standard values on individual pesticides. This process must take pesticide mixtures into account when developing toxicological and regulatory standards.
➢ Development of protective agents against pesticide-induced oxidative stress should be among the toxicological standards sponsored by regulatory agencies.

Hopefully, the highlighted recommendations could make the present study more significant regarding public health, agriculture practice, pesticide management, and global regulatory jurisdictions.

## Figures and Tables

**Table 1:** Joint action analysis for the mixture of methomyl and abamectinbased on biochemical data measurements^@^ in the plasma of treated male rats.

Biochemical Parameter	Control Value (C)	MET Value (A)	ABM Value (B)	MET+ABM Value (M)	Interaction Index (I.I.)	Joint Action
MDA ^††^	1.35	2.22	2.36	2.71	0.89	An
nmol/mL						
SOD ^†^	104.87	86.23	81.85	72.71	1.06	An
µmol/min/mL						
CAT ^††^	0.48	0.61	0.63	0.85	1.07	Po
µmol/min/mL						
GPx ^†^	0.87	0.73	0.76	0.69	1.05	Ad
µmol/min/mL						
GR ^†^	85.37	66.01	58.80	48.73	1.07	An
nmol/min/mL						
GST ^†^	1.07	0.85	0.85	0.80	1.10	An
µmol/min/mL						
CYP_450_^†^	0.14	0.04	0.04	0.03	2.13	An
nmol/min/mL						
T ^†^	4.86	1.83	1.99	1.69	1.71	An
ng/mL						
T_4_^†^	3.89	2.37	2.41	1.83	1.20	An
µg/dL						

^@^Data refer to Figure 1, Figure 2 and Figure 3 and each value is the mean of eight values.

Biochemical parameter abbreviations: MDA: malondialdehyde; SOD: superoxide dismutase; CAT: catalase; GPX: glutathione peroxidase; GR: glutathione reductase; GST: glutathione-S-transferase; CYP_450_: cytochrome P_450_; T: testosterone; T_4_: thyroxine. Interaction Index (I.I.) = (M + C)/(A + B) ^†^ Case of negative effect. ^††^ Case of positive effect. Joint action: An = antagonism; Po = potentiation; Ad = additive.

**Table 2:** The percent of change in some biochemical parameters, related to oxidative stress and hormonal disturbance, in male rats induced by methomyl (MET), abamectin (ABM), andthe mixture (MET+ABM), and the ameliorative effect of zinc supplementation.

Treatment	Biochemical Parameters								
	MDA	SOD	CAT	GPx	GR	GST	CYP_450_	T	T_4_
	nmol/mL	µmol/min/mL	µmol/min/mL	µmol/min/mL	nmol/min/mL	µmol/min/mL	nmol/min/mL	ng/mL	µg/dL
Control (a)	1.35	104.87	0.48	0.87	85.37	1.07	0.136	4.86	3.89
Methomyl									
MET (b)	2.22	86.23	0.61	0.73	66.01	0.85	0.038	1.83	2.37
MET+Zn (c)	1.68	98.81	0.56	0.89	72.95	0.92	0.122	3.25	3.15
% of Change *	64.44	−17.77	27.08	−16.09	−22.68	−20.56	−72.06	−62.35	−39.07
Ameliorative Index **	1.24	0.94	1.17	1.02	0.85	0.86	0.90	0.67	0.81
Abamectin									
ABM (b)	2.36	81.85	0.63	0.76	58.80	0.85	0.037	1.99	2.41
ABM+Zn (c)	1.62	96.18	0.55	0.88	83.55	0.93	0.118	3.42	3.17
% of Change *	74.81	−21.95	31.25	−13.8	−31.12	−20.56	−72.79	−59.06	−38.05
Ameliorative Index **	1.20	0.92	1.15	1.0	0.98	0.87	0.87	0.70	0.82
Methomyl+Abamectin									
MET+ABM (b)	2.71	72.71	0.85	0.69	48.73	0.80	0.027	1.69	1.83
MET+ABM+Zn (c)	1.92	85.55	0.62	0.82	71.68	0.82	0.083	2.78	2.92
% of Change *	100.74	−30.67	77.08	−20.69	−42.92	−25.23	−80.15	−65.23	−52.96
Ameliorative Index **	1.42	0.82	1.29	0.94	0.84	0.77	0.61	0.59	0.75

Data refer to Figure 1, Figure 2 and Figure 3 and each value is the mean of eight values. * % change in the biochemical parameter in question (effect of pesticide) = [(b − a)/a] × 100; ** Amelioration Index (AI) (effect of zinc co-administration) = c/a; Biochemical parameter abbreviations: MDA: malondialdehyde; SOD: superoxide dismutase; CAT: catalase; GPX: glutathione peroxidase; GR: glutathione reductase; GST: glutathione-S-transferase; CYP_450_: cytochrome P_450_; T: testosterone; T_4_: thyroxine.
